# How environmental stressors affect reproductive potential in a saltmarsh plant species *Plantago maritima*

**DOI:** 10.1002/ece3.7277

**Published:** 2021-03-06

**Authors:** Magdalena Lazarus, Jacek Mazur, Katarzyna Wszałek‐Rożek, Adrian Zwolicki

**Affiliations:** ^1^ Department of Plant Taxonomy and Nature Conservation Faculty of Biology University of Gdańsk Gdańsk Poland; ^2^ Department of Vertebrate Ecology and Zoology Faculty of Biology University of Gdańsk Gdańsk Poland

**Keywords:** active protection, brackish saltmarshes, germination, grazing, mowing, phenotypic plasticity, rooting, seed mass, trampling

## Abstract

We examined whether the presence or absence of different environmental stressors influenced the reproductive potential of a saltmarsh species—*Plantago maritima*. We focused on total seed output, seed quality, and biomass of progeny. So far, there are no studies trying to answer the question of how different saltmarsh management affects the quality of seed in saltmarsh species. For the purposes of the study, plots subjected to light mowing, light or heavy grazing, trampling, or rooting were designated in three nature reserves in Poland. On each plot, the abundance of infructescences per sq. meter was calculated. Mature infructescences were collected, and their length and number of fruit capsules were measured. The seeds obtained from fruit capsules were weighted and sown in controlled conditions. The germination rate and the final germination percentage were calculated. A representative number of sprouts were grown. After a period of 2 months, the individuals of *P. maritima* were harvested and their total dry mass was measured. It was found that heavy grazing had the greatest effect on all of the studied characteristics. The presence of this factor resulted in shorter infructescences with a smaller number of fruit capsules. However, this phenomenon was compensated by the higher abundance of infructescences per sq. meter. At the same time, seeds produced by grazed individuals were significantly lighter. Interestingly, intensive trampling by people affected *P. maritima* individuals in a similar way to heavy grazing, while mowing and rooting had less impact on the considered characteristics. Although a positive correlation between seed mass and germination success was found, the altogether lower seed mass had a negligible effect on germination parameters. Also, the differences in seed parameters did not affect dry mass of obtained progeny grown in laboratory conditions. Synthesis and applications: Different environmental stressors, such as grazing and mowing, have an effect on reproductive potential of a saltmarsh species *P. maritima*. In the case of habitats created anthropogenically, such as brackish saltmarshes, the role of management is crucial for their conservation. Therefore, searching for the best active protection methods is important. In light of the results obtained, extensive or rotational grazing appears to be the best form of saltmarsh management.

## Graphical abstract

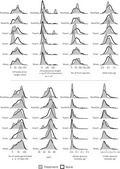

## 1 INTRODUCTION

Phenotypic variation of species can be environmentally (phenotypic plasticity) and/or genetically (genetic differentiation) induced (e.g., Bossdorf et al., 2005; Bradshaw, 1965; Scheiner et al., 2007; Sultan & Bazzaz, 1993). Phenotypic plasticity, recognized as a major source of phenotypic variation (Sultan, 2004), allows organisms to survive through the possibility of producing different functionally appropriate phenotypes by an individual genotype (Pigliucci et al. 2006) or changing its phenotypic state or activity as a response to variations in environmental conditions (Garland & Kelly, 2006). This ability to adapt is considered to be particularly valuable in the case of species that grow in places where environmental conditions change over relatively short periods of time or are spatially patchy (Miner et al., 2005). Phenotypic plasticity should be favored by natural selection, provided that genetic variation is available (Smekens & Tienderen, 2001). However, it can signify not only potential benefits, but also potential costs.

Species from the *Plantago* genus are known for their phenotypic plasticity (e.g., Blom, 1983 and references therein; van Hinsberg & van Tienderen, 1997; Smekens & van Tienderen, 2001), which increases their survival chances under different environmental conditions (Sultan, 2000). This also applies to *Plantago maritima* (sea plantain), the morphological variability of which includes the clump size, leaf area, and number and length of flower spikes (Blom, 1992; Jerling, 1988).

*Plantago maritima* is a wind‐pollinated, self‐incompatible perennial herb (Dinnetz & Jerling, 1997). As it was found for other species of the *Plantago* genus, its pollen is not transported over long distances and is dispersed within a few meters (Bos et al., 1984). Gene flow is also restricted by the distance to which the seeds can be transported. For *P. maritima*, it is not more than several dozen centimeters (Jerling, 1988). Moreover, a sticky covering of the seeds attaches them to the ground or vegetation and restricts their further mobility (Dinnetz & Jerling, 1997). According to the studies, although *P. maritima* produces viable seeds (79%), they are almost absent from the persistent seed bank (Hutchings & Russell, 1989; Jerling, 1983; Ungar, 2001). The seed bank for this species is transient and reflects the seed output of the current vegetation. Vegetative propagation is rare (van Damme, 1992; Dinnetz & Jerling, 1997; Nilsson & Ågren, 2006).

In Europe, *P. maritima* is a common component of coastal saltmarshes, which, as ecosystems, play an important role on different levels: as habitats for plants and animals, in erosion control, water purification, carbon sequestration etc. (e.g., Barbier et al., 2011; Valiela et al., 2002). Along the Baltic coast, saltmarshes with their brackish nature are man‐made and therefore need sustainable management in the form of mowing and/or livestock grazing, preferably extensive (e.g., Bakker et al., 1997; Bos et al., 2002; Dijkema, 1990). This kind of management in most places is no longer economically viable so an active conservation approach is needed to preserve coastal saltmarshes. However, in Poland in many cases this protection is either not implemented or implemented ineffectively due to the high cost of regular management practices and lack of social support, especially from landowners. As a result of cessation of use, the participation of large glycophytes in phytocoenosis increases (Esselink et al., 2000) and they start to limit the access of light to lower levels of vegetation (Bakker et al., 1985). Such conditions are one of the stress factors (Jerling & Liljelund, 1984) for some halophytes, including *P. maritima*, making it impossible to survive or forcing them to start investing more in biomass in order to win the race to the light (Piotrowska, 1988). Also, for those species, in a dense and high vegetation the probability of their seedling survival is very low (Bakker & de Vries, 1992). On the other hand, on intensively grazed sites, the pressure from glycophytes is relatively low, but the individuals of *P. maritima* need to survive grazing and trampling.

The saltmarshes along the Baltic coast in Poland, being variable habitats, express all the aforementioned factors. These habitats represent abandoned pastures/meadows, and extensively and intensively grazed/mowed saltmarshes. They are in some cases visited regularly by wild boars. Also, some plots along local paths are intensively trampled by tourists.

Intraspecific variation in mean seed size of a plant species is well documented (e.g., Agren, 1989; Lord, 1994; McKee & Richards, 1996; Mehlman, 1993). At the same time, the seed mass influence on germination and seedling establishment has been proven by many researchers (e.g., Bonfil, 1998; Khan, 2004; Milberg & Lamont, 1997; Seiwa & Kikuzawa, 1991). Since individuals of *P. maritima* differ morphologically, therefore, the analysis of the influence of saltmarsh management on *P. maritima* seed mass was an interesting subject. In our research, we tried to answer the question of whether and to what extent the presence or absence of environmental stressors, such as mowing, grazing, and rooting affects the reproductive potential of the sea plantain, understood as a number of seeds produced by a plant that are capable of germinating.

Since the role of management is crucial for the conservation of the plants species especially in habitats created anthropogenically such as saltmarshes, the answer to this question would enable the introduction of even better methods of active protection of *P. maritima*.

The aim of the study was to compare the influence of different environmental stressors on reproductive potential of *P. maritima*, including morphophysiological traits of its seeds and biomass of the progeny seedlings.

## 2 MATERIALS AND METHODS

### 2.1 Study sites

**FIGURE 1:**
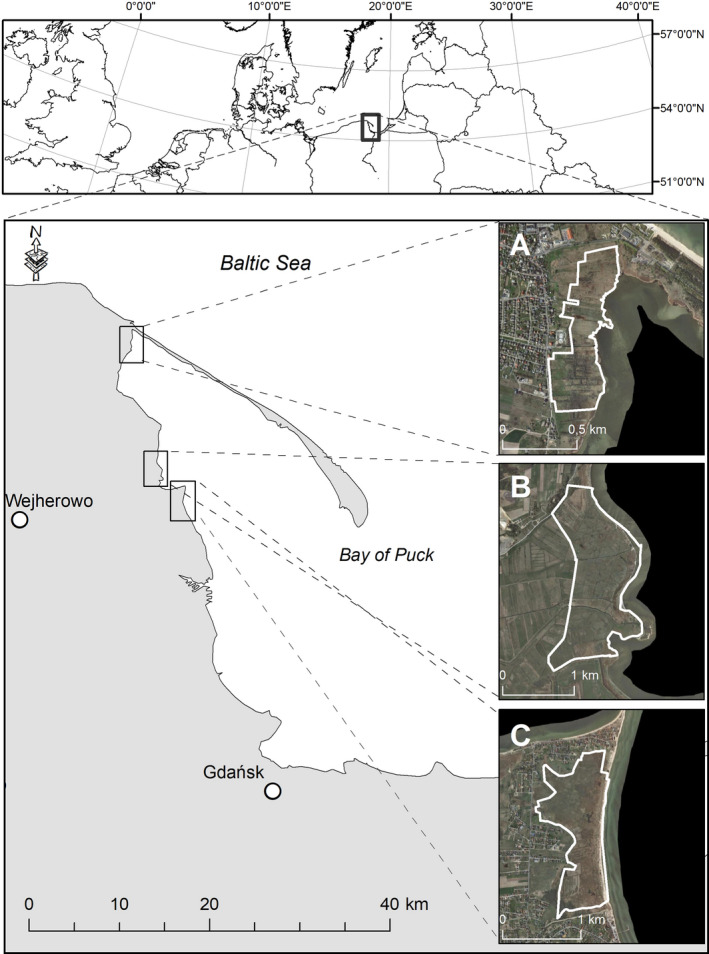
Study site location. (a) “Słone Łąki” nature reserve, (b) “Beka” nature reserve, (c) “Mechelińskie Łąki” nature reserve

The material was collected from three nature reserves in Northern Poland: “Słone Łąki,” “Beka,” and “Mechelińskie Łąki” (Figure 1). “Słone Łąki” is a nature reserve designated in 1999 for the protection of halophilous meadows with their flora and fauna, although only a small part of the reserve is regularly mowed. In “Beka,” the area was freely grazed by cattle until 1980. The nature reserve was established in 1988, but until the 1990s, almost 90% of the marshes were overgrown by reed rush as a result of a failure to implement an active conservation plan. Mowing and grazing were successfully resumed there from 1999. Currently, the stocking rate is around 1 livestock unit/ha/year (Błaszkowska et al., 2008). Both rotational grazing by cattle and horses, and the animals’ preferences for certain plant species result in a combination of heavily and lightly grazed plots. Pastures are mowed at least once a year (in autumn) or more frequently depending on reed coverage. “Mechelińskie Łąki” nature reserve was designated in 2000, although there is no active form of conservation implemented in the reserve area and saltmarshes are slowly being overgrown by rush communities.

Although the study sites differ in terms of the presence or absence and intensity of management, they are all similar as far as the main habitat factors including soil saltiness, inundation period, and inundation frequency are concerned. The potential influence of differences in soil parameters (ground water level, its pH and salinity, and soil moisture) on the results was tested with variation partitioning and was excluded.

For this reason, in the latter part of the work, the study plots were analyzed in terms of type of impact, and not place of occurrence.

### 2.2 Data sampling

Samples were collected during the first week of September 2016. We selected a set of 54 plots (1 × 1 m) that differed in management type: sites lightly mowed—mowed once/twice a year (MowL); lightly grazed—sites avoided by cattle, rarely grazed with higher vegetation (GrazL); heavily grazed—sites preferred by cattle with short vegetation (GrazH); heavy trampled—sites along the pathways intensively used by tourists (TramH); old marks of wild boar rooting—uneven surface, but almost completely overgrown by plants (RootOld); and fresh marks of rooting—uneven surface with a significant share of bare soil (RootNew). Some plots were subjected to more than one type of management, which was later taken into account in the statistical analysis. Infructescence density per one sq. meter was calculated on every plot.

Additionally, 254 infructescences (Table 1) were collected randomly from the plots, stored in envelopes, and air‐dried for 2 weeks. After drying, the length of each infructescence was measured with a resolution of 1 mm and the number of fruit capsules was counted.

**TABLE 1:** Number of samples collected and chosen for germination and growing experiment

Management type^a^	No. of plots	No. of infructescences collected	No. of infructescences used to extract seeds for germination experiment	No. of seeds used for germination	No. of sprouts used to grow seedlings
MowL	16	59	38	1,223	41
GrazL	11	39	18	528	23
GrazH	11	45	21	604	39
TramH	5	24	22	669	17
RootOld	20	67	48	1,472	55
RootNew	5	20	16	474	12
Total	54^b^	254	163	4,970	187

^a^ GrazH, heavy grazing; GrazL, light grazing; MowL, light mowing; RootNew, fresh marks of rooting; RootOld, old marks of rooting; TramH, heavy trampling.

^b^ Some plots were subjected to more than one management type.

The average length of infructescences was multiplied by their number per 1 sq. m in order to estimate the potential for seed production per site (seed yield).

### 2.3 Germination test and growth experiment

From each infructescence, a total of 30 randomly chosen seeds were weighed with a resolution of 1/100 mg. The seeds were subjected to stratification at 5°C for 60 days, then planted in a growth chamber in germination tanks on filter paper. The germination regime was set with a 25°C light(16 hr)/20°C dark (8 hr). Light was supplied from warm white fluorescent tubes. The constant high moisture of the substrate was sustained with deionized water as it is known that *P. maritima* seeds germinate best in distilled water (Lotschert, 1970). The effects of germination—the number of emerging sprouts and their condition—were checked daily for a period of 14 days as it was determined that this period was enough for an average seed to germinate completely, and after that period, almost no new sprouts appeared. The seeds were considered to have germinated when the radicle and two cotyledons were present. Mean germination time (MGT) was calculated by using the equation: MGT = ∑ (*n* × *d*)/*N*, where *n* = number of seeds germinated on each day, *d* = number of days from the beginning of the test, and *N* = total number of seeds germinated at the termination of the experiment (Ellis & Roberts, 1981).

After germination, a representative number of sprouts (Table 1) for every variant of management were grown in a separate pots filled with organic soil. After a period of 2 months, just before the plants started allocating material to reproductive organs, the individuals were harvested, and separated into underground and aboveground parts, and their dry mass was measured.

### 2.4 Statistical analysis

To test the influence of the environmental stressors on each of the response variables, eight multivariate linear regression models were performed. The statistical significance level of all regression coefficients and models was established as *α* = 0.05. All of the independent variables were coded binary, in which “1” represents the influence of a particular stressor and “0” the absence of a particular stressor. In some cases, the response variables were influenced simultaneously by more than one stressor (by two: in 39 cases by both MowL and GrazH, in four cases by MowL and RootOld, in four cases by MowL × RootNew, in four cases by GrazH × RootOld, and in four cases by GrazL × RootNew; and by three: in four cases by MowL × GrazL × RootNew); therefore, all the stressors were tested simultaneously in one linear model.

Depending on the skewness, the response data were normalized by logarithm log(*x* + 1), square root, or square transformation (compare Table 2). To describe the importance of the environmental stressor influence, the percentage of each explained variation was calculated. Statistical analysis was performed in R (R Core Team, 2017), and data visualizations were performed using “ggplot2” (Wickham, 2016) and “ggridges” packages (Wilke, 2018) in R studio (RStudio Team, 2015). The correlations between measured variables were tested with Pearson's coefficient.

**TABLE 2:** Effects of management type on different characteristics of *Plantago maritima*

Variable	Estimates/Variation	Regression coefficients/Management type^a^							Model statistics		
		Int	MowL	GrazL	GrazH	TramH	RootOld	RootNew	*R*‐sq(adj)	*F*	*p*
Infructescence length (mm)	Est.	82.83***^b^	7.72	−21.26***	−54.10***	−39.33***	3.76	−5.02	0.48	43.37	<0.001
	Var. (%)	‐‐‐	1.13	2.48	32.25	12.54	0.34	0.16			
Infructescence length* number of infructescences on 1 m^2^	Est.	2,545.28	734.15	−1111.96	−37.12	3,441.60	−51.70	5,413.69**	0.08	2.11	0.063
	Var. (%)	‐‐‐	0.19	0.09	0.65	3.84	0.74	10.62			
sqrt(number of fruit capsules)	Est.	8.16***	1.03 **	−1.41**	−3.11***	−2.16***	0.63 **	−0.009	0.41	33.56	<0.001
	Var. (%)	‐‐‐	2.47	2.54	26.09	9.99	1.44	2.47			
Mean seed mass (g)	Est.	0.61***	0.09 *	−0.01	−0.20***	−0.07*	0.08 *	−0.06	0.20	12.27	<0.001
	Var. (%)	‐‐‐	4.82	0.17	12.40	1.45	2.69	0.53			
Number of seeds germinated after 14 days (%)	Est.	62.15	−17.041 **	22.47**	−10.962	−12.35	7.12	−6.65	0.07	3.34	0.004
	Var. (%)	‐‐‐	0.40	3.21	1.40	3.07	1.73	0.46			
MGT^a^	Est.	4,345.3	294.1	−843.6*	−292.1	1,087.1***	−231.2	−1001.5**	0.15	6.40	<0.001
	Var. (%)	‐‐‐	0.19	2.58	0.59	10.42	0.01	4.20			
log(aboveground biomass (g))	Est.	−1.85	0.25	−0.20	0.18	0.27	−0.01	−0.13	<0.01	0.77	0.591
	Var. (%)	‐‐‐	0.18	0.31	0.64	1.10	0.00	0.14			
log(underground biomass (g))	Est.	2.31	−0.0002	−0.0006	0.0004	0.0008	0.0010	−0.0017	<0.01	1.03	0.408
	Var. (%)	‐‐‐	0.44	0.19	0.08	0.15	1.49	0.78			

^a^ GrazH, heavy grazing; GrazL, light grazing; MowL, light mowing; RootNew, fresh marks of rooting; RootOld, old marks of rooting; TramH, heavy trampling.

^b^ Signif. codes: “***”—0.001, “**”—0.01, “*”—0.05.

^c^ MGT, mean germination time.

## 3 RESULTS

### 3.1 The importance of management type for tested variables

Based on multivariate regression models, five of eight reproductive traits/seed quality variables of *P. maritima* were significantly influenced by the tested environmental stressors (Table 2). The most sensitive reproductive traits were as follows: infructescence length and number of fruit capsules, in which linear models explained 48% and 41% of the total variation, respectively. The following ones in the response order were variability of seed mass and MGT, in which models explained 20% and 15% variation, respectively. In case of number of seeds germinated after 14 days, the model was significant, but explained only 7% of variation. All the remaining traits (Infructescence length × Number of infructescences on 1 m^2^, above, and underground biomass of the offspring) were not significantly influenced by the management types.

The most important environmental stressor was the GrazH, reaching 32.25% of the explained variation of the infructescence length, 26.09% in case of number of fruit capsules, and 12.40% explaining seed mass variation. In terms of importance, the following one was the TramH, which was responsible for 12.54% of infructescence length variability, 10.42% of MGT, and 9.99% in case number of fruit capsules. The remaining variables in all models explained less than 5% of the total variation.

### 3.2 Differences in fruit production

**FIGURE 2:**
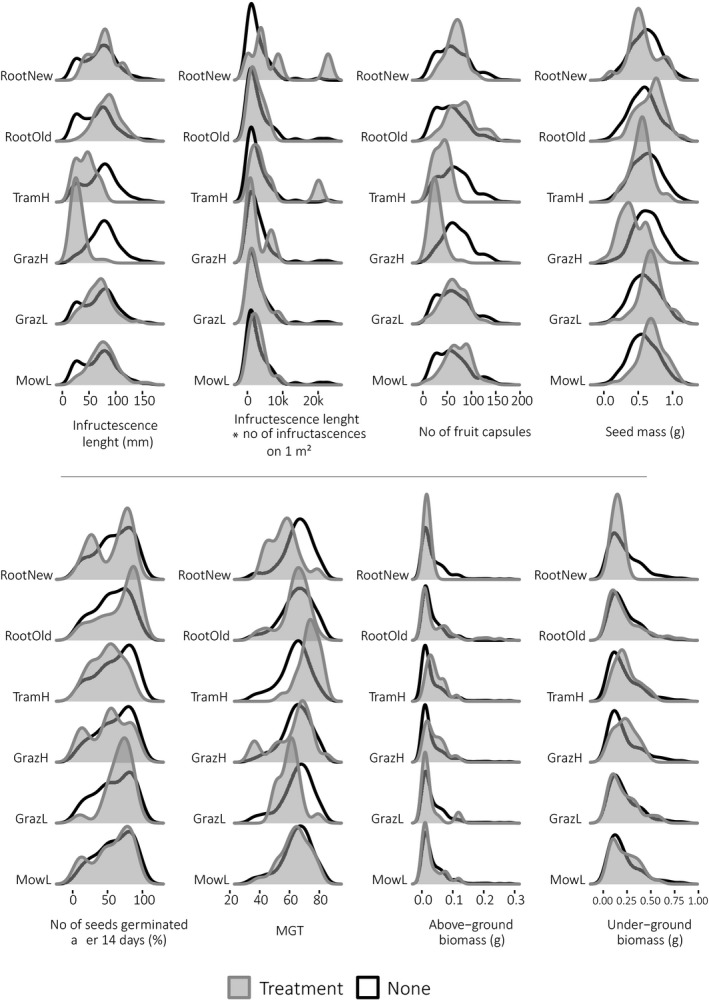
Density plots of eight tested response variables, which were influenced by six management types (gray curve) in comparisons with no treatment (black curve). GrazH, heavy grazing; GrazL, light grazing; MowL, light mowing; RootNew, fresh marks of rooting; RootOld, old marks of rooting; TramH, heavy trampling

The type of environmental stressor had a significant effect on the infructescence length of *P. maritima* (Table 2, Figure 2). Both GrazH and TramH had the strongest influence—individuals subjected to these types of land use produced the shortest infructescences in comparison with the samples collected from other plots (with an average length of 29.1 mm for GrazH and 43.5 mm for TramH). GrazL had a similar, although slightly weaker influence ($\bar{x}$ = 68.8 mm). Wild boars’ visits (both RootOld with $\bar{x}$ = 84.1 mm and RootNew with $\bar{x}$ = 75.1 mm) and MowL ($\bar{x}$ = 90.6 mm) did not significantly impact the infructescence length.

While comparing the potential for seed production between plots subjected to different kinds of treatment (the average length of infructescences multiplied by their number per 1 sq. m), we found no differences between those variables, except for the data collected for RootNew plots, for which the value obtained was higher in comparison with the remaining samples (Table 2, Figure 2).

Influences similar to those of infructescence length were observed for number of capsules (Table 2, Figure 2), with the smallest number of fruit capsules being produced on plots subjected to GrazH and TramH ($\bar{x}$ = 27.4 and $\bar{x}$ = 37.5, respectively). GrazL resulted in a slightly smaller abundance of fruit capsules being present on the infructescence axis ($\bar{x}$ = 42.4). Infructescences produced by individuals growing in both MowL ($\bar{x}$ = 69.1) and RootOld ($\bar{x}$ = 79.6) plots bore significantly more fruit capsules, while RootNew ($\bar{x}$ = 66.7) did not influence the considered variable.

### 3.3 Differences in seed mass and germination parameters

The type of environmental stressor had a significant effect on the seed mass (Table 2, Figure 2). Seeds with the smallest mass ($\bar{x}$ = 0.39 mg) were produced by the sea plantain individuals subjected to GrazH. Surprisingly, TramH had only a slight negative impact on the considered variable with the average mass of a single seed being equal to 0.54 mg. GrazL ($\bar{x}$ = 0.68 mg) and RootNew plots ($\bar{x}$ = 0.53 mg) had no influence on the seed mass, whereas individuals growing in RootOld ($\bar{x}$ = 0.67 mg) and MowL ($\bar{x}$ = 0.69 mg) plots produced slightly heavier seeds in comparison with the remaining samples.

Despite all the differences in seed material collected from the plots subjected to different environmental stressors, we found that the type of environmental stressor did not affect the number of seeds germinated after 14 days. The only exceptions were MowL, which resulted in a significantly lower percentage of germination (55.9%) after a fortnight, and GrazL, with a significantly higher percentage of germination (67.0%) at the end of experiment (Table 2, Figure 2).

MGT was shorter in the case of seeds collected from RootNew and GrazL plots, and longer for those gathered from individuals subjected to TramH. The remaining kinds of treatment did not influence MGT for the seeds of *P. maritima* (Table 2, Figure 2).

### 3.4 Differences in offspring

The fact that the sowing material was collected from individuals subjected to different kinds of environmental stressors had no influence on the above‐ and belowground biomass of the offspring individuals grown from the gathered seeds (Table 2, Figure 2).

### 3.5 Relationship between tested variables

**FIGURE 3:**
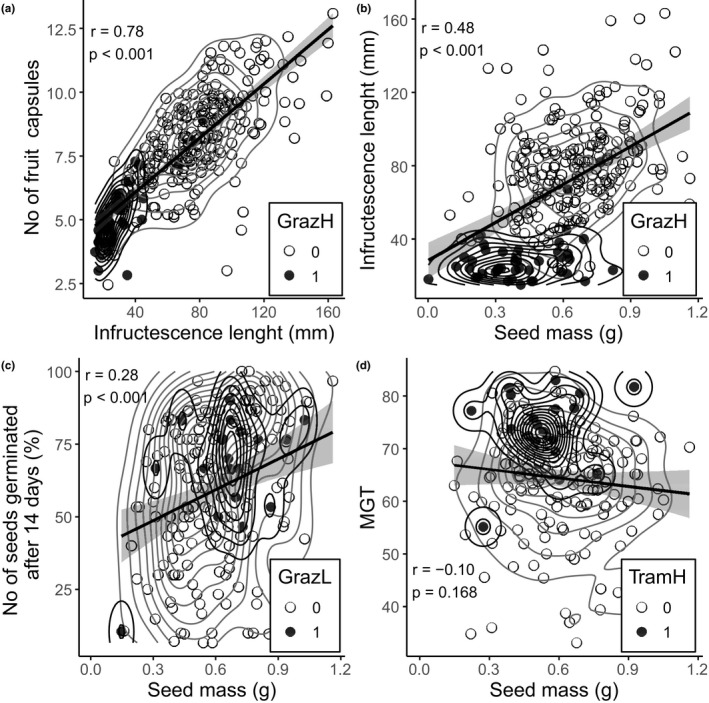
Scatter plots with two‐dimensional density functions presenting the relationship between selected response variables, with fitted linear regressions. The gray circles represent selected management types (1), while the empty ones represent no treatment (0). GrazH, heavy grazing; GrazL, light grazing; *r*, Pearson's correlation coefficient; TramH, heavy trampling. The gray area along regression lines describes confidence intervals

We found a strong positive correlation between the length of infructescence and the number of fruit capsules (*r* = 0.76, *p* < 0.001) (Figure 3a). Also, seed mass was positively correlated with the infructescence length (*r* = 0.45, *p* < 0.001) (Figure 3b). Also, there was a positive correlation between seed mass and the number of seeds germinated after a 14‐day period of incubation (*r* = 0.28, *p* < 0.01) (Figure 3c). The lighter were the seeds, the less of them germinated within the fortnight, although this correlation was weak. However, seed mass did not affect their MGT (*r* = −0.10, *p* = 0.168) (Figure 3d).

## 4 DISCUSSION

### 4.1 Reproductive traits

The results obtained in our research prove that the length of infructescences is impacted by the type of environmental stressor, wherein the influence of grazing (especially GrazH) was the strongest of all of the considered factors. Individuals subjected to GrazH had the shortest infructescences (only 1/3 the length of the samples from ungrazed sites). It is commonly known that cattle feed on *P. maritima* (Jensen, 1985). Jerling and Andersson (1982) found that the loss of flowers of this plant species due to grazing was severe throughout the whole flowering season. On intensively grazed marshes, longer shoots have an increased risk of being grazed and trampled during reproduction, so only short infructescences are favored.

Trampling by people along walking routes had a similar effect to grazing (TramH). The stress caused by trampling is a sufficient factor influencing *P. maritima* morphology, regardless of being eaten by animals on pastures.

The production of longer infructescences by individuals not grazed or trampled does not have to be connected only with the absence of a factor causing mechanical damage. One cannot forget that beyond any other influence, on brackish saltmarshes, grazing limits the development of glycophytes. After the cessation of grazing, the race for light begins, plants invest their energy to outgrow other competitors, and this results in taller vegetation (Adam, 1990; Bakker & de Vries, 1992).

The lack of significant differences in the infructescence length between samples collected from plots with old and fresh marks of wild boar rooting can be explained by the fact that rooting is generally not a permanent factor. If the freshly rooted marshes become overgrown within a short time, the influence on plant individuals is not visible.

Although the strong influence of the type of environmental stressor on the infructescence length was discussed above, the analysis of the infructescence length in relation to their number per 1 sq. meter shows that smaller seed production by shorter infructescences is compensated by their higher abundance. This kind of compensation in response to grazing can be observed in different plant species (e.g., Paige, 1992; Paige & Whitham, 1987). It stands in contrast to the conclusion drawn by Jerling and Andersson (1982) that grazing, which causes heavy loss of *P. maritima* flower spikes, must greatly reduce the production of seeds.

On nongrazed saltmarshes, although the infructescences produced by sea plantain individuals are long, the number of flower shoots per one square meter is lower. This can be related to the generally smaller number of sea plantain individuals and to the fact that a lack of light in denser vegetation prevents this species from flowering (Jerling & Andersson, 1982).

### 4.2 Influence of site management on seed quality

An average seed mass for *P. maritima*, which was 0.61 mg, had an intermediate value in relation to those known from literature (according to Bakker and de Vries (1992)—0.53 mg and according to Jerling (1988) for central marsh—0.79 mg and high marsh—0.83 mg). However, the seed mass clearly varied depending on the place of harvest.

A positive correlation between infructescence length and seed mass was found. Shorter infructescences collected from GrazH marshes had, in general, lighter seeds. This may be due to the fact that sea plantain individuals growing on grazed plots had to produce infructescences in the course of the season in order to replace those that had been grazed and therefore did not have an opportunity to allocate too much energy into seeds to improve their quality. With the necessity to replace flower spikes that were grazed comes the possibility that the fruits that were collected during this research developed mostly from late flowers. Therefore, it can be presumed that the time it takes to produce mature seeds that are ready for dispersal may also affect the seed mass. The aforementioned assumptions would be in accordance with Jerling and Andersson's (1982) discovery that, in the case of *P. maritima*, grazing affects the relative proportion of late and early flowers, wherein earlier flowers produce significantly heavier seeds.

It should be mentioned here that although TramH resulted in *P. maritima* producing relatively short infructescences, it did not have such a strong influence on the seed mass itself. Also, GrazL had no such effect on the seed mass as GrazH, whereas a positive influence of MowL on the *P. maritima* seed mass can be explained by better development conditions for the sea plantain under this type of management, which allows plants to invest more energy in the next generation.

A relationship between seed mass and its germination parameters has been recognized by different researchers (e.g., Dolan, 1984; Dunlap & Barnett, 1983; Larsen & Andreasen, 2004; Schaal, 1980; Susko & Lovett‐Doust, 2000; Swanborough & Westoby, 1996; Weis, 1982; Zimmerman & Weis, 1983). Higher seed mass may be associated with a higher quantity of food reserves, which is particularly important for seedling establishment (e.g., Baker, 1972; Harper, 1977; Mazer, 1989).

Dinnetz and Jerling (1997) found that, for *P. maritima*, seed mass was positively correlated with germination rates. However, those authors tested differences in seed quality between individuals of different sex morphs. Their results indicated that there was no support for any superiority of females compared with hermaphrodites. In our study, it was found that there was an influence of the seed mass on the germination parameters.

There was no correlation between seed mass and MGT, although seeds collected from TramH sites germinated more slowly in comparison with others and those collected from RootNew and GrazL germinated faster. However, considering all of the results presented, it can be concluded that there may be other factors influencing the germination rates that were not analyzed during the research rather than the seed mass itself. Similarly, although the type of management did not affect the germination success (the number of seeds germinated after a 14‐day period of incubation), a positive correlation between seed mass and germination success was found, which was also proved by Dinnetz and Jerling (1997). Heavier, better‐provisioned seeds are thought to have a superior chance of establishing as seedlings in a population (Grime et al. 1988; Khan, 2004). This is important for species such as *P. maritima*, for which the possibility of seed dispersal is very poor (Jerling, 1985), its seeds germinate easily, and therefore, their contribution to a seed bank is very small (Jerling, 1983; Ungar, 2001). However, the sea plantain's chances of seedling survival are related to vegetation density and height, as well as litter accumulation, and are better overall in grazed or mowed rather than abandoned marshes (Bakker & de Vries, 1992; Bazely & Jefferies, 1986; Jerling, 1981, 1984; Jerling & Andersson, 1982; Ungar, 1998). Production of larger seeds may be an adaptation of individuals growing in abandoned pastures. It was proved that large *P. maritima* seeds produced significantly taller seedlings (Gregor, 1948), which can give them a better chance of survival in high and dense vegetation.

### 4.3 Influence of seed quality on seedling growth performance

Likewise for other plant species (e.g., Houssard & Escarré, 1991; Reich et al. 1994), it was found that the differences in seed parameters and seedling development did not influence progeny performance. The sowing experiment carried out in laboratory conditions indicated that the seed mass itself had no effect on the aboveground and belowground biomass of the offspring individuals. The result obtained in this study may indicate that after the germination period, in the field conditions the variation of descendants is influenced more by habitat or random factors than the mass of the seed from which they grew.

## 5 CONCLUSIONS

The role of management is crucial for the conservation of the plants species especially in habitats created anthropogenically such as saltmarshes. Our study shows that the type of saltmarsh management affects the reproductive potential of *P. maritima*, wherein the most influential was heavy grazing, which resulted in shorter infructescences and smaller seed mass produced by individuals subjected to this kind of treatment. Light grazing and light mowing had little effect on the tested features, while rooting turned out to be almost irrelevant.

The sea plantain adopts different strategies depending on the conditions in which it grows. The plants can produce numerous short infructescences or long infructescences but in lower numbers per unit of area; nevertheless, the total number of fruit capsules, and indirectly total seed output, is similar. However, shorter infructescences bear lighter seeds and lighter seeds are potentially less successful in germination than heavier seeds.

Although in laboratory conditions the differences in seed mass were not reflected either in the germination success or MGT, considering the restricted gene flow between *P. maritima* populations, long‐term selection may lead to genetic differentiation between heavily grazed and ungrazed populations. This, in turn, may result in the loss of adaptive abilities to the changing environment, for example, in the case of a change in saltmarsh management. This is especially important if one considers the lack of a persistent seed bank for *P. maritima* in the soil.

In the case of brackish saltmarshes, where grazing is considered to be the best management practice for *P. maritima* survival, extensive or rotational grazing that leads to a mosaic of closely grazed and lightly grazed patches turns out to be the best form of saltmarsh management.

## CONFLICT OF INTEREST

The authors declare no conflict of interest.

## AUTHOR CONTRIBUTIONS

**Magdalena Lazarus:** Conceptualization (equal); data curation (lead); formal analysis (equal); funding acquisition (lead); investigation (supporting); methodology (lead); project administration (lead); resources (lead); software (equal); supervision (lead); validation (lead); visualization (supporting); writing – original draft (lead); writing – review and editing (equal). **Jacek Mazur:** Investigation (equal); methodology (supporting); writing – review and editing (supporting). **Katarzyna Wszałek‐Rożek:** Conceptualization (equal); investigation (equal); methodology (supporting); writing – review and editing (supporting). **Adrian Zwolicki:** Formal analysis (equal); software (equal); visualization (lead); writing – original draft (supporting); writing – review and editing (equal).

## Data Availability

Dataset file of this study is available from the Dryad Digital Repository (https://datadryad.org/stash/dataset/doi:10.5061/dryad.08kprr51c).
